# Differentiating novel coronavirus pneumonia from general pneumonia based on machine learning

**DOI:** 10.1186/s12938-020-00809-9

**Published:** 2020-08-19

**Authors:** Chenglong Liu, Xiaoyang Wang, Chenbin Liu, Qingfeng Sun, Wenxian Peng

**Affiliations:** 1 School of Medical Instrument and Food Engineering, University of Shanghai for Science and Technology, Shanghai, 200093 China; 2 College of Medical Imaging, Shanghai University of Medicine and Health Sciences, Shanghai, 201318 China; 3 Department of Radiology, Ruian People’s Hospital, Zhejiang, 325200 China; 4 Department of Radiation Oncology, Chinese Academy of Medical Science (CAMS) Shenzhen Cancer Hospital, Shenzhen, 518116 China; 5 Infectious Disease Department, Ruian People’s Hospital, Zhejiang, 325200 China

**Keywords:** Machine learning, Novel coronavirus pneumonia, General pneumonia, Chest CT

## Abstract

**Background:**

Chest CT screening as supplementary means is crucial in diagnosing novel coronavirus pneumonia (COVID-19) with high sensitivity and popularity. Machine learning was adept in discovering intricate structures from CT images and achieved expert-level performance in medical image analysis.

**Methods:**

An integrated machine learning framework on chest CT images for differentiating COVID-19 from general pneumonia (GP) was developed and validated. Seventy-three confirmed COVID-19 cases were consecutively enrolled together with 27 confirmed general pneumonia patients from Ruian People’s Hospital, from January 2020 to March 2020. To accurately classify COVID-19, region of interest (ROI) delineation was implemented based on ground-glass opacities (GGOs) before feature extraction. Then, 34 statistical texture features of COVID-19 and GP ROI images were extracted, including 13 gray-level co-occurrence matrix (GLCM) features, 15 gray-level-gradient co-occurrence matrix (GLGCM) features and 6 histogram features. High-dimensional features impact the classification performance. Thus, ReliefF algorithm was leveraged to select features. The relevance of each feature was the average weights calculated by ReliefF in n times. Features with relevance larger than the empirically set threshold T were selected. After feature selection, the optimal feature set along with 4 other selected feature combinations for comparison were applied to the ensemble of bagged tree (EBT) and four other machine learning classifiers including support vector machine (SVM), logistic regression (LR), decision tree (DT), and K-nearest neighbor with Minkowski distance equal weight (KNN) using tenfold cross-validation.

**Results and conclusions:**

The classification accuracy (ACC), sensitivity (SEN), specificity (SPE) of our proposed method yield 94.16%, 88.62% and 100.00%, respectively. The area under the receiver operating characteristic curve (AUC) was 0.99. The experimental results indicate that the EBT algorithm with statistical textural features based on GGOs for differentiating COVID-19 from general pneumonia achieved high transferability, efficiency, specificity, sensitivity, and impressive accuracy, which is beneficial for inexperienced doctors to more accurately diagnose COVID-19 and essential for controlling the spread of the disease.

## Background

Since the first COVID-19 case was discovered in 2019, more than 9.47 million cases of novel coronavirus pneumonia have been diagnosed worldwide, with 484,249 deaths recently according to World Health Organization Coronavirus disease (COVID-2019) situation report − 158. Currently, the detection of COVID-19 mainly relies on nucleic acid testing. However, many infected patients with obvious typical symptoms passed multiple nucleic acid tests but diagnosed positive in the last test [1]. The high false-negative rate results in delayed treatment and even aggravating the spread of the pandemic. On February 5, National Health Commission of the People’s Republic of China launched the “Novel Coronavirus Pneumonia Diagnosis and Treatment Program (Trial Version 5)”, which updated the diagnostic criteria for novel coronavirus pneumonia with adding CT imaging examinations as one of the main basics for clinical diagnosis of COVID-19. CT screening is considerably popular, easy to operate and sensitive to COVID-19, which is critical for both early diagnosis and pandemic control.

Nevertheless, influenza virus pneumonia and other types of pneumonia might occur in this season as well. In some aspects, especially according to clinical features, it is troublesome to differentiate COVID-19 from general pneumonia. For instance, the main manifestations of COVID-19 in the early stage were fever, fatigue, dry cough, and expiratory dyspnea while patients with general pneumonia have similar symptoms [2]. COVID-19 pneumonia places a huge burden on the health care system because of its high morbidity and mortality. Therefore, early diagnosis and isolation of GP patients and COVID-19 patients can better prevent the spread of the pandemic and optimize the allocation of medical resources. However, except for the overlapping symptoms and detection abnormalities, CT manifestations of GP and COVID-19 were similar, causing instability and uncertainty for distinguishing them [3, 4].

**Fig. 1:**
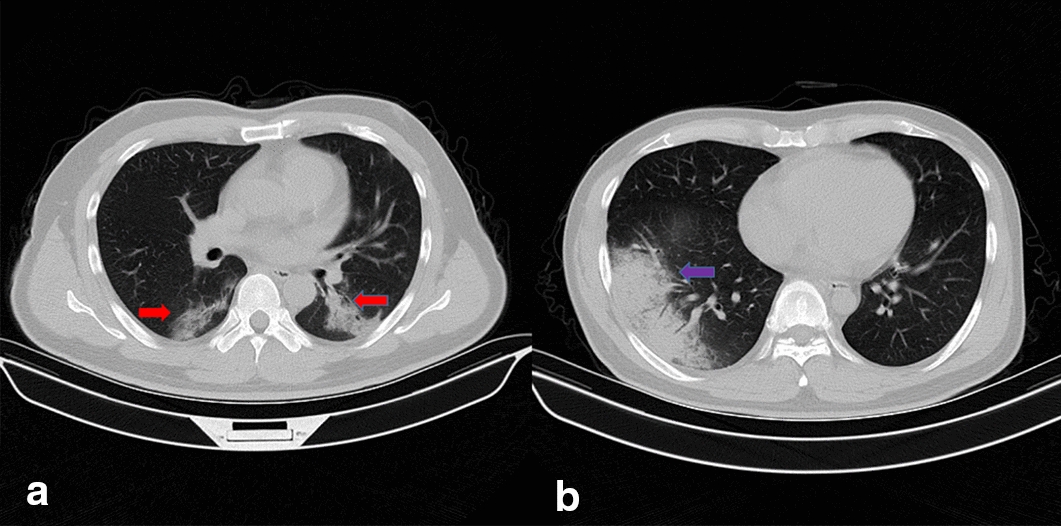
Samples of COVID-19 and GP CT images. Picture** a** is the CT image of COVID-19 with bilateral GGOs while picture** b** is the CT image of GP with unilateral GGO. The red arrows point at the GGOs of COVID-19 and the blue arrow points at the GGO of GP

Typical CT manifestations of COVID-19 patients consist of pleural indentation sign, unilateral or bilateral pulmonary ground-glass opacities, opacities with rounded morphology and patchy consolidative pulmonary opacities with the predominance in the lower lung [5–8]. GP infections have similar CT manifestations at presentation. However, COVID-19 presents more bilateral extensive GGO while GP shows more unilateral GGO or consolidation [9]. Furthermore, the other CT findings of GP and COVID-19 are difficult to observe and the areas of lungs contain large scale of insignificant extraneous parts. To avoid interference from irrelevant information and more accurately and stably identify COVID-19 from GP, GGO was cropped as the ROI and features were extracted based on ROIs. Figure 1 shows the samples of COVID-19 and GP CT images from the collected dataset.

Lin et al. proposed a deep learning model, COVNet, based on visual features from volumetric CT images to distinguish COVID-19 from community acquired pneumonia [10]. 4536 three-dimensional CT images (COVID-19: 30%; community acquired pneumonia: 40%; non-pneumonia: 30%) were included in their study. U-net was applied to crop the lung region as the ROI and both 2D and 3D features were extracted by COVNet based on the ROIs. Then the features were combined and inputted to the proposed scheme for predictions. The sensitivity and specificity for detecting COVID-19 were 90% and 96% while for CAP were 87% and 92%. The AUCs were 0.96 and 0.95. However, the features learned by deep learning models are embedded in a network of millions of weights. Thus, the method lacks interpretability and transparency.

Charmaine et al. evaluated ResNet with a location-attention mechanism model for screening COVID-19 [11]. Two ResNet models were enrolled in their study. Three-dimensional features were extracted by ResNet-18 and fed into ResNet-23 with location-attention mechanism in the full-connected layer for classification while ResNet without location-attention mechanism was applied as well for comparison with the proposed method. Accordingly, the results show the proposed method achieved better performance with an overall accuracy of 86.7%.

Asif et al. proposed CoroNet model based on Xception architecture using X-ray images to differentiate COVID-19 from heathy, bacterial pneumonia and viral pneumonia [12]. Notably, Xception is a transfer learning model which pertained to ImageNet dataset and then retained on the collected X-ray dataset. In the proposed architecture, the classical convolution layers were replaced by convolutions with residual connections. The overall accuracy was 89.6% while average accuracy of detecting COVID-19 was 96.6%. To test the stability and robustness, CoroNet was evaluated on the dataset prepared by Ozturk et al. [13] with an accuracy of 90%.

Ozturk et al. developed DarkNet model based on the you only look once (YOLO) system to detect and classify COVID-19 [13]. Their model achieved the accuracy of 98.08% for classifying COVID-19 and non-infections and 87.02% for distinguish COVID-19 from no-findings and GP. Nevertheless, the proposed methods by Asif et al. and Ozturk et al. were based on X-ray images. X-ray screening is not sensitive to GGOs which is one of the most significant manifestations at the early stages of COVID-19. This can cause high error rate and ineffective containment of the pandemic.

Kang et al. developed a machine learning method with structured latent multi-view representation learning to diagnose COVID-19 and community acquired pneumonia [14]. In their work, V-Net was leveraged to extract lung lesions. Then, radiomic features and handcrafted features, totally 189-dimensional features, were extracted from the CT images. In the end, the proposed model yielded the best accuracy, which was 95.50%. The sensitivity and specificity were 96.6% and 93.2%. Compared with other methods in the study, the accuracy was improved by 6.1–19.9% and the sensitivity and specificity were improved by 4.61–21.22%.

To our knowledge, most recent researches carried out for detecting COVID-19 are based on deep learning. However, deep learning models require a large scale of training data while initially the COIVD-19 samples are in shortage. Transfer learning might be promising method in terms of small amount of data while negative transfer may exist, for initial dataset and target domains may not relate to each other and the standards on what types of training data are sufficiently related are not clear.

Machine learning plays an unsubstitutable role in artificial intelligence with outstanding results in medical imaging classification. We developed a machine learning method using ensemble of bagged tree based on statistical texture features of CT images, particularly focusing on differentiating COVID-19 from GP, demonstrating high efficiency in the identification of COVID-19 and GP, helping to reduce misdiagnosis and control pandemic transmission.

## Material

From January 2020 to March 2020, there were 73 COVID-19 cases confirmed by nucleic acid test positive and 27 general pneumonia cases enrolled in this study (age ranges from 14 to 72 years). Both COVID-19 and GP patients who had undergone chest CT scans were retrospectively reviewed by two senior radiologists. Of the COVID-19 cases, 12 patients without obvious characteristics on CT images were excluded (negative rate 16.4%, 12/73). Finally, 61 confirmed COIVD-19 cases and 27 general pneumonia cases were enrolled in this study.

The images were independently assessed by two radiologists. If the radiologists disagreed with each other, a senior radiologist would be invited to review the pulmonary CT images and make the final examination. All the CT images were generated from the Siemens Sensation 16-layer spiral CT (Siemens, Erlangen, Germany). The image format was Digital Imaging and Communications in Medicine (DICOM). The scan parameters were: tube voltage 120 kV; tube current automatic regulation; 1-2 mm cross-sectional thickness; 1–2 mm cross-sectional distance; scan pitch 1.3; and 16 × 0.625 mm collimation.

## Results

The proposed diagnosis method is ensemble of bagged trees based on feature combination 5 (*T* = 0.11) including ROI delineation, feature extraction, feature selection and classification which are explicitly described in “Method” section. In this section, the results of feature selection, effectiveness of optimal feature combination 5 compared to original features, and comparison of EBT algorithm and four other classification methodologies are described. The experimental result demonstrated that the proposed COVID-19 diagnosis method outperformed other methods in terms of accuracy, sensitivity, specificity and AUC.

### Results of feature selection

**Fig. 2:**
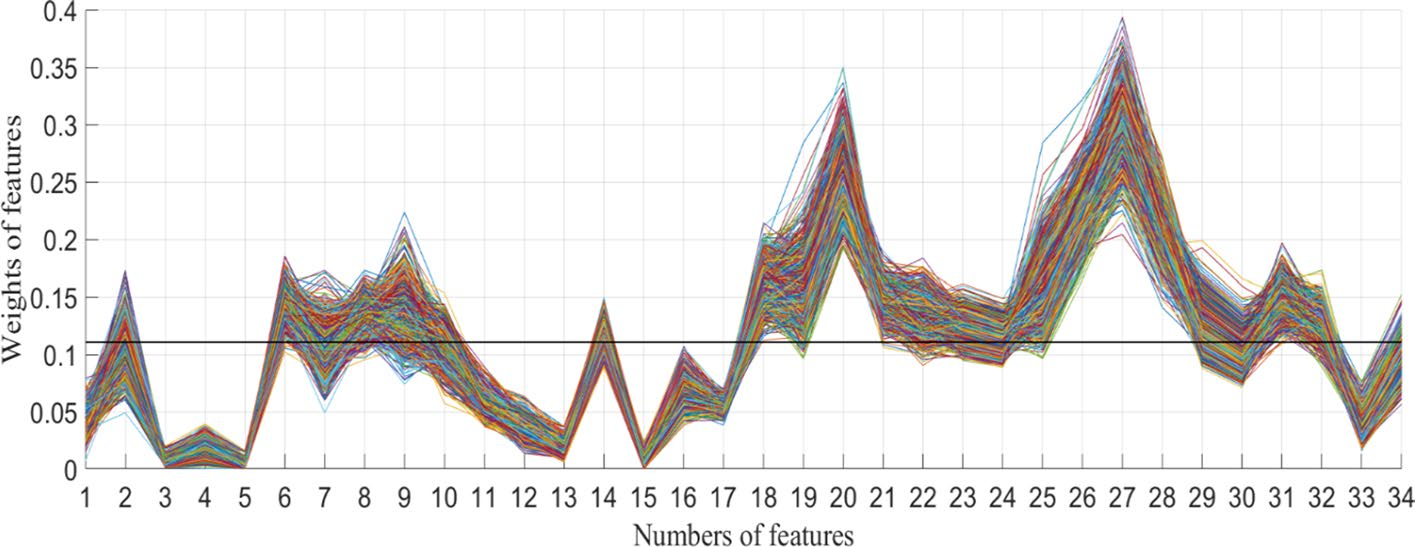
The weight curves of 34 features based on ReliefF algorithm. The *X-*axis represents the numbers of features. The *Y* axis represents the weights of different features at different times. The algorithm run 1000 times represented by curves with different colors. The dark straight line represents weight = 0.11, which is the proposed threshold *T*

Table 1 and Fig. 2 show the relevance of each feature and weight curves of each feature based on ReliefF algorithm. In order to select the optimal feature combination, the proposed threshold *T* was set to 0.11. To justify optimization, combination 1 (*T* = 0.11*), combination 2 (*T* = 0.12), and combination 3 (*T* = 0), combination 4 (*T* = 0.10) were considered to compare with combination 5 (*T* = 0.11). Features included according to four different *T* values are shown in Table 2 (the corresponding feature names of the feature numbers are presented in Table 4 in “Method” section).

**Table 1:** Relevance of each feature based on ReliefF algorithm

Feature	Relevance	Feature	Relevance	Feature	Relevance	Feature	Relevance	Feature	Relevance
1	0.0421	8	0.1329	15	0.0043	22	0.1348	29	0.1250
2	0.1036	9	0.1338	16	0.0687	23	0.1257	30	0.1025
3	0.0050	10	0.1059	17	0.0548	24	0.1170	31	0.1490
4	0.0178	11	0.0582	18	0.1604	25	0.1574	32	0.1250
5	0.0032	12	0.0360	19	0.1574	26	0.2267	33	0.0389
6	0.1434	13	0.0163	20	0.2561	27	0.2977	34	0.0963
7	0.1036	14	0.1184	21	0.1469	28	0.2094

**Table 2:** Selected features of four combinations

Combination	*T*	Feature numbers
1	0.11^a^	1,2,3,4,5,7,10,11,12,13,15,16,17,30,33,34
2	0^b^	All 34 features
3	0.10	2,6,7,8,9,10,14,18,19,20,21,22,23,24,25,26,27,28,29,30,31,32
4	0.12	6,8,9,18,19,20,21,22,23,25,26,27,28,29,31,32
5	0.11	6,8,9,14,18,19,20,21,22,23,24,25,26,27,28,29,31,32

^a^Select features with relevance smaller than the threshold *T*

^b^No feature selection was applied

### Performance evaluation

**Fig. 3:**
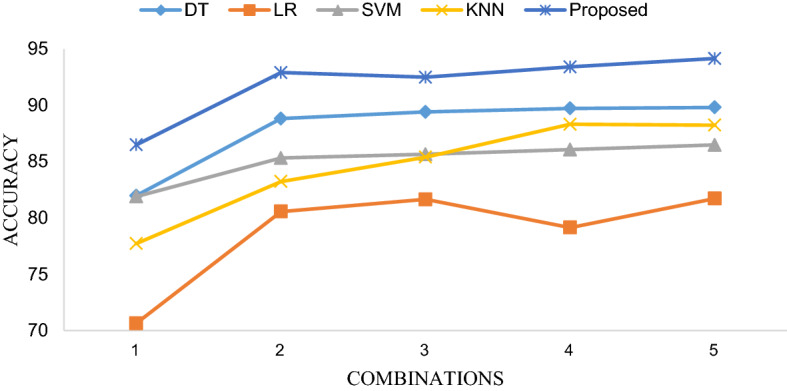
Accuracy comparison of five classifiers with different feature combinations

**Fig. 4:**
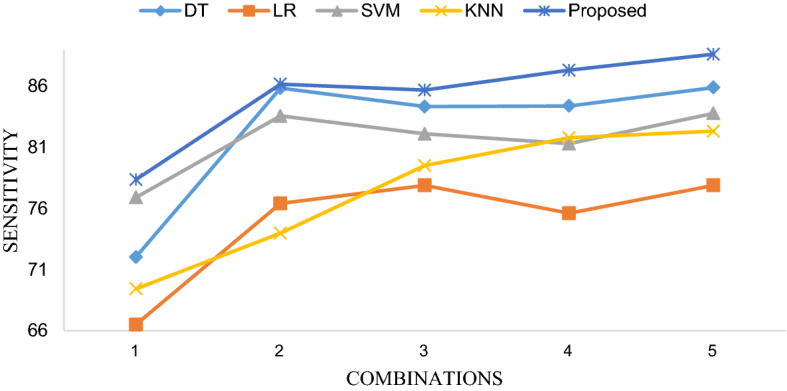
Sensitivity comparison of five classifiers with different feature combinations

**Fig. 5:**
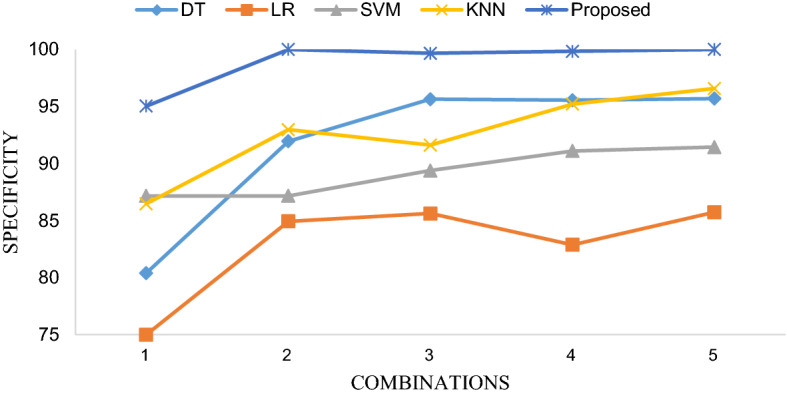
Specificity comparison of five classifiers with different feature combinations

**Fig. 6:**
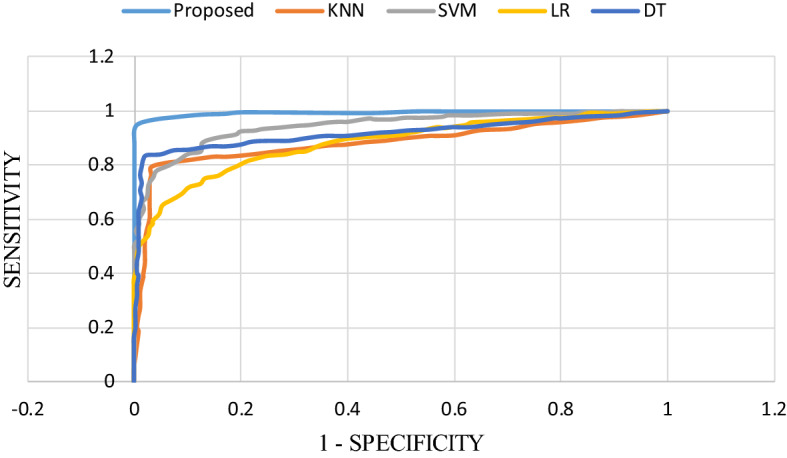
Comparison of receiver operating characteristic curves for the proposed classifier, KNN, SVM, LR, and DT using feature combination 5. The receiver operating characteristic curves for the proposed EBT models had an AUC that was significantly greater than that for four other models

Table 3 shows the diagnosis performance of 5 classifiers based on 5 different feature combinations. In order to intuitively present the differences in accuracy, sensitivity and specificity of different methods using different feature combinations, we visualized them with line Fig. 3, line Fig. 4 and line Fig. 5, respectively. The receiver operating characteristic (ROC) curves of EBT algorithm and 4 other classifiers using the optimal feature combination 5 are presented in Fig. 6.

**Table 3:** Diagnosis performance based on different methods using different combinations

Method	Combination 1	Combination 2	Combination 3	Combination 4	Combination 5
DT					
ACC (%)	81.98	88.82	89.41	89.73	89.82
SEN (%)	72.03	85.85	84.34	84.39	85.90
SPE (%)	80.40	91.95	95.64	95.55	95.69
LR					
ACC (%)	70.64	80.57	81.65	79.15	81.73
SEN (%)	66.50	76.42	77.89	75.61	77.89
SPE (%)	75.00	84.93	85.62	82.88	85.73
SVM					
ACC (%)	81.90	85.32	85.65	86.07	86.48
SEN (%)	76.91	83.58	82.11	81.30	83.79
SPE (%)	87.16	87.16	89.38	91.10	91.44
KNN					
ACC (%)	77.73	83.23	85.40	88.32	88.24
SEN (%)	69.43	73.98	79.51	81.79	82.33
SPE (%)	86.47	92.97	91.61	95.21	96.58
EBT					
ACC (%)	86.49	92.91	92.49	93.41	94.16
SEN (%)	78.37	86.18	85.69	87.32	88.62
SPE (%)	95.03	100.00	99.66	99.83	100.00

#### Effectiveness of optimal feature combination 5 compared to original features

Figure 3 elucidates that five classifiers using feature combination 5 achieved the highest accuracy than that obtained by other feature combinations. The measurements in the X-axis ranging from 1 to 5 represents the sequence numbers of the feature combinations in Table 2. Figures 4 and 5 substantiate that the sensitivity and specificity of the optimal feature set outperformed that of combination 2 as well as combination 1, 3 and 4. To be noted, combination 2 contains 34 features which indicates that no feature selection was applied, which illustrates that feature selection is essential.

#### Comparison of EBT and four other classification methodologies

As shown in Table 3, the best result was obtained by EBT algorithm with feature combination 5, leading to accuracy, sensitivity and specificity of 94.16%, 88.62% and 100.00%, respectively. The three line figures reveal that EBT algorithm achieved clearly better performance compared with other classification methodologies using no matter what feature combinations. Figure 6 demonstrates ROC curves of five models based on feature combination 5. And the AUCs (area under curve, AUC) of DT, LR, SVM, KNN and EBT are 0.91, 0.88, 0.94, 0.88, and 0.99, respectively. The EBT provided the best AUC. Therefore, the promising results validate that the proposed method can accurately and robustly differentiate COVID-19 from GP.

## Discussion

The proposed diagnosis method was evaluated in terms of accuracy, sensitivity and specificity. As shown in Eqs. 2–4 in “Method” section, accuracy measures the ability of the diagnosis system to correctly detect COVID-19 and GP. Sensitivity demonstrates the proportion of correctly classified COVID-19 cases. Specificity illustrates how good the method is at identifying GP cases. As shown in Table 3, the highest accuracy, sensitivity and specificity achieved by EBT algorithm with feature combination 5 were 94.16, 88.62, and 100.00, respectively. It shows that the proposed method did better performance in detecting GP than COVID-19. To alleviate class imbalance, we did data augmentation on GP images. However, data augmentation techniques cannot increase the diversity of GP features. Although the proposed method achieved the specificity of 100.00%, which suggests no GP cases were erroneously classified, there is no denying that it has the probability of over-fitting caused by shortage in GP images.

CT of COVID-19 infections presents consolidation, GGO, pulmonary fibrosis, interstitial thickening, and pleural effusion in both lungs [15–17] while CT of GP infections presents multifocal nodular opacity with a surrounding halo, diffuse patchy GGO, interlobular septal thickening, multiple ill-defined nodules and consolidation in both lungs [18]. Thus, most resent researches have proposed heterogeneous methods based on the whole lung region. For example, Wang et al. developed COVID-19Net for diagnosing COVID-19 with automatic lung segmentation of CT images using DenseNet121-FPN [19]. Notably, DenseNet121-FPN is also a transfer learning framework, which was pre-trained on ImageNet dataset as well. The sensitivity and specificity of the method were 78.9% and 89.93% in the training set. In the two validation sets, the sensitivities were 80.39% and 79.35% and the specificities were 76.61% and 81.16%. As mentioned previously in the background section, the deep learning method proposed by Lin et al. implemented U-net for lung segmentation [10]. It achieved the sensitivity and specificity of 90% and 96%. Zhang et al. used AI system with a two-stage segmentation framework to segment lung lesions and then diagnose COVID-19 [20]. The first stage of the segmentation framework was manual annotation and the second stage was DeepLabv3-based backbone for lung lesion segmentation. In their work, they achieved smoother and clearer boundaries compared with experts. Besides, they validated their system in the dataset from outside China with 84.11% accuracy, 86.67% sensitivity, and 82.26% specificity for differentiating COVID-19 from GP. Wu et al. developed a multi-view deep learning fusion model based on the architecture of ResNet50 with threshold segmentation and morphological optimization algorithms for lung segmentation [21]. The accuracy, sensitivity and specificity of their model in the testing set were 0.760, 0.811 and 0.615, respectively. However, compared with these studies, we did GGO segmentation instead of lung lesion segmentation. Our proposed machine learning method in combination with GGO segmentation accomplished an accuracy of 94.16% for distinguishing COVID-19 from GP. It also has a high sensitivity and specificity of 88.62% and 100.00%, respectively. Therefore, we achieved better performance in diagnosing COVID-19 based on only GGOs. The results empirically validate that COVID-19 and GP can be robustly classified based on GGOs.

Despite the remarkable performance of the proposed methods, limitations still exist in our study. First of all, the ROIs were manually delineated which is rather time-consuming especially when doctors are racing against time to save lives. Also, GGOs were the exclusive segmented features of CT images of COVID-19 and GP and spending more time on ROI segmentation is apparently unworthy while the whole lung region contains irrelevant or even pernicious information for diagnosis. Hence, further study should be processed on automatically and preciously detect and segment ROIs without manual help. Finally, our established model did not determine which specific general pneumonia it was, such as viral or bacterial, mainly due to insufficient data. More data will be collected and the prognosis of GPs will be considered in our future study.

## Conclusions

This study explored an ensemble of bagged tree algorithm with statistical textural features for differentiating novel coronavirus pneumonia from general pneumonia. The classification accuracy, sensitivity, and specificity of our proposed method yield 94.16%, 88.62% and 100.00%, respectively. It is noteworthy that compared with four other machine learning classifiers, EBT achieved consistent better performance. The results show that classifiers with feature selection excelled classifiers without feature selection by 1–5% for accuracy, 2–10% for sensitivity and 0–4% for specificity. More importantly, classifiers with feature selection take shorter time. Therefore, feature selection is beneficial for promoting the diagnosis of COVID-19 in terms of all evaluation indexes.

Furthermore, GGOs were proved to play a significant role in distinguish COVID-19 from GP, which provide reference opinions for radiologists to better diagnose COVID-19. And extensive experiments will be applied on more features of COVID-19 individually and unitedly in our future work. In conclusion, the experimental results show that, as compared to other state-of-the-art works, the proposed method achieved pronouncedly superior performance with a small amount of CT images.

## Methods

### Overview of the proposed diagnosis framework

**Fig. 7:**
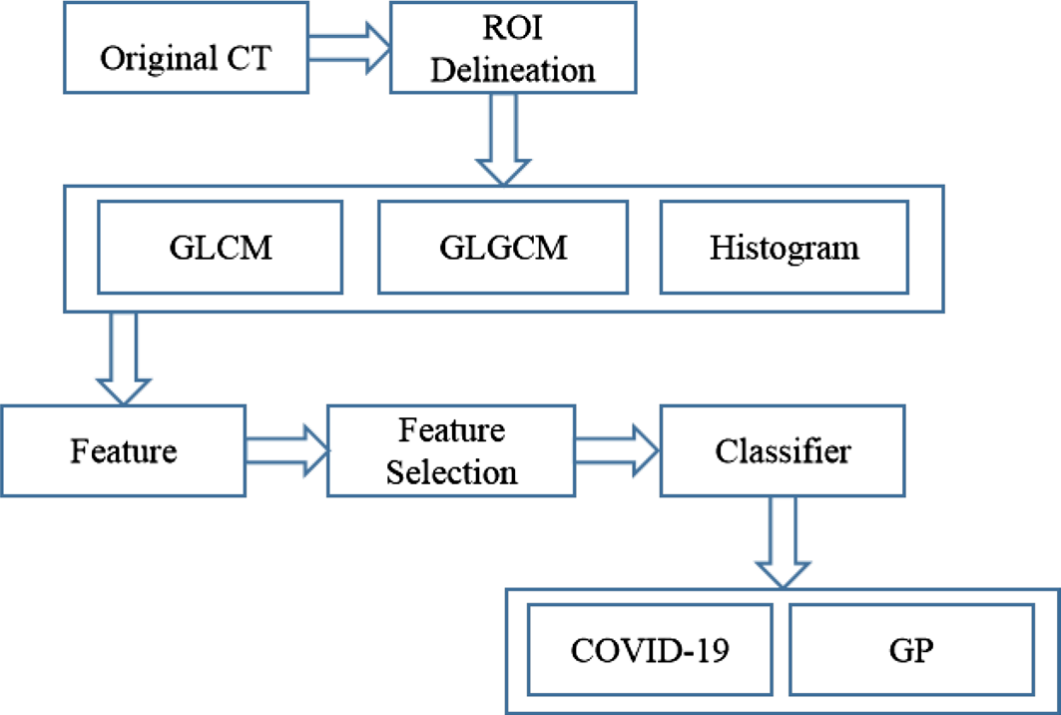
The flowchart of the proposed diagnosis framework

Machine learning algorithms integrated with statistical textural features are leveraged to differentiate COVID-19 from GP. Figure 7 illustrates the block diagram of the proposed diagnosis framework. After data collection, to more accurately extract features of COVID-19 and GP, manual delineation of the ROIs were performed based on GGOs. The details of ROI delineation are presented in “Delineation of ROIs” section. In the next step, 34 statistical texture features including 13 GLCM features, 15 GLGCM features and 6 histogram features were extracted from the ROIs. After that, ReliefF algorithm was used to select features for time-saving and avoiding over-fitting. As a result, five feature combinations remained while combination 5 with 18 features were classified as the proposed feature group. Details are described in the following feature selection and results part. In the last stage of diagnosis process, the selected features with labels were combined and input to five classifiers while the ensemble of bagged tree is the proposed algorithm for classification. Five classifiers with five feature combinations, respectively, were evaluated in term of accuracy, specificity, sensitivity and AUC.

The framework consists of 4 major steps: delineation of ROIs, feature extraction, feature selection, and classification. Each of the steps is described in detail in the following parts of this paper.

### Delineation of ROIs

To improve the accuracy of the diagnosis method, precise segmentation of the ROIs from irrelevant parts was essential for feature extraction. Thus, GGO region, which is the main CT manifestations, was taken as ROI. The software of MRIcro 1.4 was used to extract the rectangle ROI of COVID-19 and GP. ROIs were delineated in CT images based on aforementioned GGOs. The main processes of ROI delineation are as follows: (1) a rectangular region as large as possible, which is the ROI, was delineated within GGOs and export the whole image with delineation to a PNG image; (2) PNG images were binarized to get the ROI boundary and fill the rectangular region to get the ROI template; (3) the ROI templates were used to extract the ROI in the original DICOM image; (4) the gray level of the ROI image was converted to 256 gray levels and the images were resized to 32 × 32 pixels. Consequently, 615 COVID-19 and 146 GP ROIs were cropped. It is apparent that COVID-19 images were four times larger than GP images while imbalanced data cannot reflect the true distribution of two categories, which could affect the classification performance. Thus, we rotated the GP images by 90°, 180°, 270°. Ultimately, the number of GP images was augmented to 584. In conclusion, 1199 ROI images were enrolled for feature extraction.

### Feature extraction

In this stage, a total of 34 statistical texture features were extracted from the ROI images of COVID-19 and GP as shown in Table 4, which contain 13 GLCM [22] features, 15 GLGCM [23] features and 6 histogram [24] features. GLCM and GLGCM are the predominant second-order statistical texture analysis methods to characterize the features of an image, which have been widely applied in medical image processing [25, 26]. Besides, GLCM considers the statistical and spatial relationship of the pixels in the image. It is created by calculating how often pairs of pixel with specific values and in a specified spatial relationship occur in an image. Then 13 statistical texture features are extracted based on the grey-level co-occurrence matrix. In contrast with GLCM, GLGCM captures not only gray-scale features, but also the second-order statistics of gray-level gradients while gradients indicate the information of image edge which provides significant features of an image. In addition, the histological characteristics of COVID-19 and GP can be well reflected in the gray mode, and the gray histogram is an intuitive statistical method [27]. It is a one-dimensional function of the gray level and belongs to the first-order statistical method. After obtaining all texture feature data, due to the different calculation methods of each feature, the numerical value changes in a wide range. Therefore, to facilitate calculation, all data are normalized to [0, 1] based on their respective dimensions, the normalized equation (1) is as follows:

1 $$X* = \left( {X - {\text{IN}}} \right) / \left( {{\text{MAX}} - {\text{MIN}}} \right) ,$$

where *X* is the original data of the *N*_th_ dimension, MIN is the minimum value in the *N*_th_ dimension, MAX is the maximum value in the *N*_th_ dimension, X^*^ is the normalized feature.

**Table 4:** Description of extracted features

Feature groups	Description
GLGCM	1. Little gradient advantage; 2. Large gradient advantage; 3. Gray heterogeneity; 4. Gradient heterogeneity; 5. Energy; 6. Average gray; 7. Average gradient; 8. Gray mean square error; 9. Gradient mean square error; 10. Correlation; 11. Gray entropy; 12. Gradient entropy; 13. Hybrid entropy; 14. Inertia; 15. Inverse difference moment
GLCM	16. Angular second moment; 17. Correlation; 18. Entropy; 19. Contrast; 20. Inverse difference moment; 21. Sum average; 22. Sum entropy; 23. Sum variance; 24. Variance; 25. Dissimilarity; 26. Inertia; 27. Difference variance; 28. Difference entropy
Histogram	29. Entropy; 30. Uniformity; 31. Mean intensity; 32. Standard deviation; 33. Kurtosis; 34. Skewness

### Feature selection

Feature selection plays a critical role in enhancing the performance of medical imaging classification. High-dimensional features cause over-fitting, lower accuracy, comprehension difficulty and it is rather time-consuming. Thus, feature selection is leveraged to select a subset of features, which makes the evaluation criteria reach the optimal level, from the original feature set. ReliefF algorithm is classified as a typical filter method for feature selection [28]. It calculates the weight for each feature based on the capability to identify feature value differences between nearest neighbor instance pairs. The weight of a random given feature decreases if the difference of the feature value is observed in the nearby instance of the same class (called nearest hit). Alternatively, the weight of a random given feature increases if the difference of the feature value is observed in the nearby instance of the difference class(called nearest miss). ReliefF searches for *k-*nearest hits and misses and averages their contribution to the weights of each feature [29]. Furthermore, *m* random features will be selected and the algorithm repeated n times to improve reliability. After n iterations, divide the sum of each feature’s weights by *n*. This is noted as the relevance. Features with relevance greater than a threshold *T* are selected. Therefore, different thresholds yield different combinations. Generally, *T* is supposed to be greater than 0, for negative weights means negative impact on classification.

### Feature classification

The ensemble of bagged tree, which is a supervised classification scheme, is regarded as the proposed classification algorithm [30]. It adopts the idea of bootstrap aggregating to enhance the stability and increase the accuracy. The training data are partitioned into several subsets by random selecting with replacement. Each subset is trained to construct independent base models. All the predictions from different models are applied to majority voting scheme. As a result, it reduces the influence of noise data and is less susceptible to over-fitting, which improves the robustness.

For comparison with the performance of the EBT algorithm, SVM, LR, DT, KNN are implemented with the same texture feature extraction methods and the same feature selection method. To superiorly identify the differences of the results, a tenfold cross-validation strategy method is adopted. In tenfold cross-validation, the original data set is equally divided into 10 subsamples. Of the 10 subsamples, 9 subsamples are used as training set while the remaining one is taken as validation set. The process is repeated 10 times until each of the 10 subsamples is utilized as validation set. The average of the 10 results is retained as the final estimation.

### Statistics

The classification metrics used included AUC, sensitivity, specificity, accuracy. Let TP (true positive) denote the number of samples belonging to class positive and correctly classified; TN (true negative) denote the number of samples belonging to class negative and correctly classified; FP (false positive) denote the number of samples not belonging to class positive but misclassified as class positive; FN (false negative) denote the number of samples not belonging to class negative but misclassified as class negative [31]. Classification accuracies are reported in terms of accuracy, sensitivity, specificity as

2 $${\text{Accuracy}} = \left( {{\text{TP}} + {\text{TN}}} \right) / \left( {\text{TP + TN + FP + FN}} \right),$$

3 $${\text{Sensitivity}} = {\text{TP / }}\left( {{\text{TP}} + {\text{FN}}} \right),$$

4 $${\text{Specificity}} = {\text{TN / }}\left( {{\text{TN}} + {\text{FP}}} \right).$$

## Data Availability

The dataset analyzed during the current study was derived from the following public domain resources: https://pan.baidu.com/s/1Ux9dpa1wtquNee4hEh1OWQ, code: k23c.

## Abbreviations

CT: Computed tomography
COVID-19: Novel coronavirus pneumonia
GP: General pneumonia
EBT: Ensemble of bagged tree
DICOM: Digital imaging and communications in medicine
kV: Kilovolt
GLCM: Gray-level co-occurrence matrix
GLGCM: Gray-level-gradient co-occurrence matrix
SVM: Support vector machine
LR: Logistic regression
DT: Decision tree
KNN: K-nearest neighbor with Minkowski distance equal weight
ROC: Receiver operating characteristic curve
AUC: Area under the receiver operating characteristic curve
TP: True positive
TN: True negative
FP: False positive
FN: False negative
